# The Chitinase-Like Protein YKL-40 Modulates Cystic Fibrosis Lung Disease

**DOI:** 10.1371/journal.pone.0024399

**Published:** 2011-09-20

**Authors:** Andreas Hector, Michael S. D. Kormann, Ines Mack, Philipp Latzin, Carmen Casaulta, Elisabeth Kieninger, Zhe Zhou, Ali Ö. Yildirim, Alexander Bohla, Nikolaus Rieber, Matthias Kappler, Barbara Koller, Ernst Eber, Olaf Eickmeier, Stefan Zielen, Oliver Eickelberg, Matthias Griese, Marcus A. Mall, Dominik Hartl

**Affiliations:** 1 Department I, Children's Hospital, University of Tübingen, Tübingen, Germany; 2 Department of Paediatrics, University of Berne, Inselspital, Berne, Switzerland; 3 Division of Pediatric Pulmonology and Allergy and Cystic Fibrosis Center, Department of Pediatrics III, University of Heidelberg, Heidelberg, Germany; 4 Translational Lung Research Center, University of Heidelberg, Heidelberg, Germany; 5 Comprehensive Pneumology Center, Institute of Lung Biology and Disease (iLBD), University Hospital, Ludwig Maximilians University and Helmholtz ZentrumMünchen, Munich, Germany; 6 Department of Dermatology and Allergy, Ludwig-Maximilians-University, Munich, Germany; 7 Department of Pediatric Pulmonology, Allergy and Cystic Fibrosis, Children's Hospital, Frankfurt, Germany; 8 Research Center, Children's Hospital, Ludwig-Maximilians-University, Munich, Germany; 9 Respiratory and Allergic Disease Division, Paediatric Department, Medical University of Graz, Graz, Austria; Johns Hopkins School of Medicine, United States of America

## Abstract

The chitinase-like protein YKL-40 was found to be increased in patients with severe asthma and chronic obstructive pulmonary disease (COPD), two disease conditions featuring neutrophilic infiltrates. Based on these studies and a previous report indicating that neutrophils secrete YKL-40, we hypothesized that YKL-40 plays a key role in cystic fibrosis (CF) lung disease, a prototypic neutrophilic disease. The aim of this study was (i) to analyze YKL-40 levels in human and murine CF lung disease and (ii) to investigate whether *YKL-40* single-nucleotide polymorphisms (SNPs) modulate CF lung disease severity. YKL-40 protein levels were quantified in serum and sputum supernatants from CF patients and control individuals. Levels of the murine homologue BRP-39 were analyzed in airway fluids from CF-like βENaC-Tg mice. *YKL-40*SNPs were analyzed in CF patients. YKL-40 levels were increased in sputum supernatants and in serum from CF patients compared to healthy control individuals. Within CF patients, YKL-40 levels were higher in sputum than in serum. BRP-39 levels were increased in airways fluids from βENaC-Tg mice compared to wild-type littermates. In both CF patients and βENaC-Tg mice, YKL-40/BRP-39 airway levels correlated with the severity of pulmonary obstruction. Two *YKL-40* SNPs (rs871799 and rs880633) were found to modulate age-adjusted lung function in CF patients. YKL-40/BRP-39 levelsare increased in human and murine CF airway fluids, correlate with pulmonary function and modulate CF lung disease severity genetically. These findings suggest YKL-40 as a potential biomarker in CF lung disease.

## Introduction

Chronic lung disease determines the morbidity and mortality of cystic fibrosis (CF) patients [1]. CF lung disease is characterized by a nonresolving neutrophilic inflammation with impaired antibacterial killing and proteolytic destruction of pulmonary tissue [2].

Humans lack chitin, but they express chitinases and chitinase-like proteins (CLP). Both chitinases and CLP belong to the 18-glycosyl-hydrolase family, including acidic mammalian chitinase (AMCase), chitotriosidase, oviductin, YKL-40 in humans, while YM-1, YM-2, AMCase, oviductin, and breast regression protein (BRP-39) have been described in mice. CLPs bind chitin, but do not have enzymatic chitinase activity due to mutations in their highly conserved putative enzyme sites [3]. The prototypical CLP YKL-40 (YKL for the first three N-terminal residues of a 40 kDa protein, also termed human cartilage glycoprotein (HcGP)-39) gene is localized on chromosome 1 [4]. The murine homologue of YKL-40 is breast regression protein of 39 kDa (BRP-39). YKL-40/BRP-39 was initially described to be expressed in cancer cells and several lines of evidence support the view that YKL-40/BRP-39 plays a role in cell proliferation, survival and tissue remodeling [5]–[7].

YKL-40 protein levels are detectable in human serum and were unexpectedly found to be significantly increased in patients with severe asthma [8] and chronic obstructive pulmonary disease (COPD) [9], two disease conditions featuring neutrophilic infiltrates. Based on these studies and a previous report indicating that neutrophils secrete YKL-40 [21], we hypothesized that YKL-40 plays a key role in CF lung disease, a prototypic neutrophilic disease.

We quantified YKL-40 protein levels in serum and airway fluids (sputum supernatants) from individuals with CF and control subjects without pulmonary diseases, assessed BRP-39 levels and lung function in a mouse model of CF-like lung disease (βENaC-Tg mice) and analyzed the impact of *YKL-40* gene variants on YKL-40 protein levels and age-adjusted CF lung disease severity.

## Materials and Methods

### Patient cohorts

In total, 338 CF patients were included in the study (see genotyping cohort below, **Methods S1** and **Table S1**). Out of this cohort we were able to collect both peripheral blood and induced sputum *ex vivo* sample material simultaneously from 59 patients in order to quantify YKL-40 protein levels. Accordingly, YKL-40 protein levels were analyzed in serum and induced sputum supernatants of patients with CF (n = 59) and healthy control subjects (n = 26) (Table 1). The CF group included 31 male and 28 female patients with a mean age of 22±15 (SD) years. Inclusion criteria were the diagnosis of CF by clinical symptoms and positive sweat tests (sweat Cl- concentration >60 mmol/l) or disease-causing mutations in the CFTR gene, forced expiratory volume in 1 second (FEV1) >30% of predicted value [31], [32] and being clinically stable and on steady concomitant therapy at least four weeks prior to the study. Genetically, 34 CF patients were ΔF508 homozygous, 19 were ΔF508 heterozygous carriers of the CFTR gene and 6 had other CFTR mutations than ΔF508.Twenty-six control subjects without pulmonary diseases were selected as the control group (12 male, 14 female; mean age: 25±9 SD years). These subjects had no suspected or proven pulmonary disease and were free of respiratory tract infections [30]. Informed written consent was obtained from all subjects included in the study or their parents, and all study methods were approved by the local ethics committee (Ethics committee of the Ludwig-Maximilians-University (LMU), Munich, Germany) and by the institutional review board of the Children's Hospital of the Ludwig-Maximilians-University, Munich, Germany. Induced sputum was obtained, processed and stored as described previously [22], [33]. Cell-free sputum supernatant was stored at −80°C until analysis.

**Table 1:** Patient groups.

	Cystic fibrosis	Controls
N	59	26
Age [yrs]	22±15	25±9
Sex (m∶f)	31/28	12/14
WBC (10^9^/l)	10±5	8±3
FEV_1_ (% pred)	63±15	-
Neutrophils in sputa (%)	83±32	17±10
*P. aeruginosa* †	35	0
Antibiotics	35	0
dF508*homozygous/heterozygous/other*	34/19/6	n.d.

Results are expressed as means ± SD; m: male, f: female; WBC: white blood count; FEV_1_: Forced expiratory volume in 1 second (% of predicted);

† *P. aeruginosa* bacteria isolated in at least 2 consecutive sputum samples with a minimum of a 6-month interval; n.d. not determined.

### Experimental animals

The generation of βENaC-Tg mice has been previously described [36]. Animal studies of the betaENaC-Tg mice (ID 6608) were approved by the Regierungspräsidium Karlsruhe or by the Regierung von Oberbayern, Munich, Germany. The colony was maintained on a mixed genetic background (C3H/HeN × C57BL/6N), and βENaC-Tg mice were identified by PCR. Wild-type littermates served as controls in all experiments. Mice were housed in a pathogen-free animal facility and had free access to chow and water. For further details please refer to the Supplementary section. For bronchoalveolar lavage (BAL) mice were deeply anesthetized via intra-peritoneal injection of a combination of ketamine/xylazine (120 mg/kg and 16 mg/kg, respectively), the trachea was cannulated and lungs were carefully lavaged twice with 800 µl PBS. BRP-39 protein levels were measured in BAL supernatant using aR&D System's ELISA. Total cell counts were determined and differential cell counts performed on cytospin preparations. Studies were performed by investigators who were blinded with respect to the genotype. We used two different invasive pulmonary function devices (both from Buxco Research Systems; Wilmington, NC): a forced maneuver system and a FinePointe RC system. Mouse pulmonary function testing was performed and analyzed as published previously [37]. All mice were anesthetized with i.p. MMF (Medetomidin, Midazolam, Fentanyl), intubated and placed in a FinePointe RC system. In a heated plethysmograph chamber, mice were ventilated at an average rate of 140 breaths per minute, and flow, mouth and esophageal pressure and heart rate were monitored to measure resistance and dynamic compliance. After an initial acclimation period of three minutes, two subsequent one-minute measurements were performed and averaged. After the resistance and compliance measures, mice were transferred to a forced pulmonary maneuvers system. Quasistatic pressure volume and fast flow volume maneuvers were run three times each and averaged to obtain forced expiratory volume at 100 ms, forced vital capacity and chord compliance values.

### ELISA

YKL-40 and BRP-39 protein levels were measured in duplicates by a commercially available, sandwich enzyme-linked immunosorbent assay (ELISA) kit (R&D Systems) according to the manufacturer's instructions.

### Genotyping cohort

Polymorphisms were genotyped in a CF population (**Tables S1 and S2**) to investigate the influence of single nucleotide polymorphisms (SNPs) [34] on CF lung disease. Informed written consent was obtained from all subjects included in the study or their parents, and all study methods were approved by the local ethics and by the institutional review board. For more details see ONLINE SUPPLEMENT.

### Statistical analysis

Differences between the patient groups were calculated using the non-parametric Kruskal-Wallis test. When a significant difference was found, the non-parametric Mann-Whitney *U* test was applied for two-group comparisons. Correlations were verified with Spearman *rho* test. Associations between SNPs and qualitative outcomes were tested by using Pearson X^2^ tests [35]. A *P* value of <0.05 was considered to be significant. A correlation was assumed when the correlation coefficient was >0.3. Statistical analysis was performed with Prism 4.0 (Graph Pad Software, San Diego, CA, USA) and STATA version 8.2 for Windows (STATA Corporation, College Station, TX, USA).

## Results

### Increased YKL-40/BRP-39 levels in human and murine cystic fibrosis airway fluids

**Figure 1:**
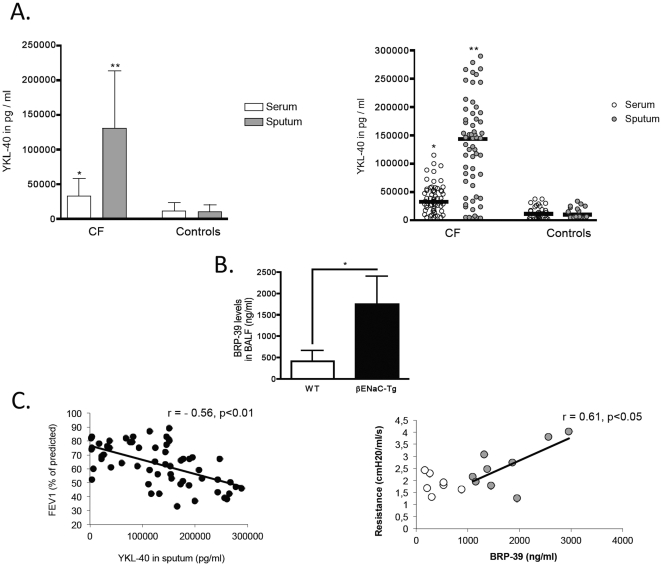
YKL-40 levels in human and murine CF lung disease. A. YKL-40 levels in CF patients and healthy controls YKL-40 protein levels were quantified by means of ELISA in sera and sputum supernatants from cystic fibrosis (CF) or healthy control subjects. The left graph depicts means ± SDs, the right scatter graph depicts individual patients with horizontal bars as medians; *P<0.05 ** *P*<0.01 disease group compared to the control group. Horizontal bars indicate comparisons among the disease groups. B. BRP-39 levels in murine CF-like lung disease. BRP-39 protein levels were quantified by means of ELISA in BAL fluids from βENaC-Tg (n = 9) and WT mice (n = 7). *P<0.05; C. YKL-40/BRP-39 airway levels and lung function in human and murine CF lung disease. Left panel: YKL-40 protein levels were quantified by means of ELISA in sputum supernatants from CF patients (n = 59). FEV1: Forced expiratory volume in 1 second (% of predicted). Right panel: BRP-39 protein levels were quantified by means of ELISA in BAL fluids from βENaC-Tg (n = 9, grey fill) and WT mice (n = 7, white fill).

YKL-40 levels were significantly increased in both sputum supernatants and serum from CF patients compared to healthy control individuals (Figure 1A). Within individual CF patients, YKL-40 protein levels were consistently higher in sputum than in serum. Notably, YKL-40 levels in CF sputa and CF sera showed a broad range of detection from levels similar to healthy control levels up to 30-fold higher levels in CF patients compared to healthy controls (Figure 1A right graph).YKL-40 serum levels correlated positively with YKL-40 sputum levels in both CF patients (r = 0.69, p<0.01) and, to a lesser extent, healthy control individuals (r = 0.42, p<0.05). Sputum levels of YKL-40 correlated positively with numbers of neutrophils in sputa (r = 0.74, p<0.01), but not with numbers of eosinophils(r = 0.13, p>0.05), lymphocytes(r = 0.18, p>0.05) or macrophages (r = 0.10, p>0.05).

Consistent with human CF lung disease, protein levels of the murine YKL-40 homologue BRP-39 were highly increased in bronchoalveolar lavage fluid (BALF) from βENaC-Tg mice compared to BALF from WT mice (Figure 1B). Both in human CF patients as well as in murine CF lung disease, YKL-40/BRP-39 airway levels correlated with airflow obstruction in pulmonary functions tests (FEV_1_ and resistance, respectively, Figure 1C), but not with parameters of pulmonary restriction (data not shown).

When viewed in combination, these studies demonstrate that YKL-40/BRP-39 levels are increased in CF patients and a mouse model of CF-like lung disease, and are associated with obstructive pulmonary function.

### *YKL-40* gene variants modulate YKL-40 levels and cystic fibrosis lung disease severity

**Figure 2:**
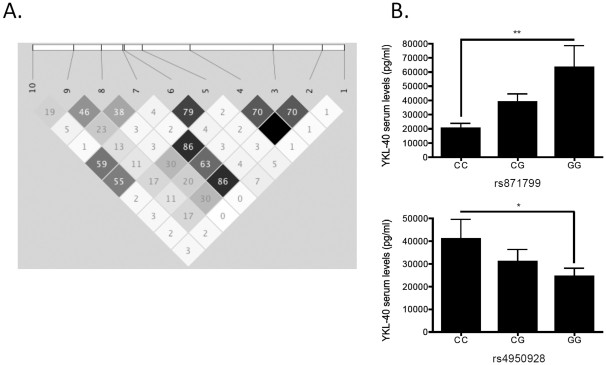
YKL-40 SNPs. A. Location and linkage disequilibrium (R^2^) of *YKL-40* polymorphisms genotyped in the CF population. B. YKL-40 serum levels in CF patients stratified for the respective rs871799 (upper panel) or the rs4950928 (lower panel) genotype. *P<0.05, **P<0.01.

Based on the finding that both YKL-40 serum and sputum levels showed a high extent of heterogeneity among CF individuals, we investigated whether *YKL-40* gene variants modulate YKL-40 serum levels and CF lung disease severity. Based on previous results from *YKL-40* SNP studies in asthmatics (11), we genotyped ten candidate SNPs tagging the *YKL-40* locus in a well-characterized CF patient cohort (**Table S2** and **Figure 2A**). Two *YKL-40* SNPs, located in the promoter (rs871799) and in the exon 5 (rs880633) region, respectively, were found to modulate age-adjusted lung function in CF patients (longitudinal FEV_1_age-adjusted at 20 years according to the method described previously by Schluchter et al. [19] (**Table 2** and **Figure 2A**). Furthermore, the promoter SNPs rs871799 and rs4950928 modulated YKL-40 serum levels (**Figure 2B**). These studies demonstrate that *YKL-40* gene variants are associated with YKL-40 serum levels and pulmonary function in CF patients.

**Table 2:** Association test results for *YKL-40* SNPs.

SNP	Position	Location	Alleles	MAF FEV_1_% @20 yrs≤70	MAF FEV_1_% @20 yrs>70	OR*	*P*value
rs871799	−14,120	Promoter	C/G	0.113	0.192	**0.24**	**0.043**
rs2153101	−12,723	Promoter	T/A	0.215	0.189	1.31	0.711
rs946263	−9,630	Promoter	A/G	0.144	0.138	0.73	1.000
rs4950929	−4,374	Promoter	T/G	0.215	0.200	0.82	1.000
rs6691378	−1,371	Promoter	G/A	0.136	0.154	1.78	0.469
rs10399805	−247	Promoter	G/A	0.156	0.178	1.11	1.000
rs4950928	−131	Promoter	C/G	0.226	0.183	1.09	1.000
rs1538372	+1,220	Intron 2	G/A	0.371	0.337	0.94	1.000
rs880633	+2,951	Exon 5	T/C	0.419	0.522	**0.46**	**0.016**
rs2275352	+5,573	Intron 7	G/A	0.188	0.154	0.71	1.000

*recessive model; MAF, minor allele frequency; FEV1%@20 yrs, age-adjusted (at 20 years) longitudinal forced expiratory volume (FEV_1_) (19); OR, odds ratio.

## Discussion

CF lung disease is characterized by a vicious cycle of neutrophilic inflammation, infection and tissue remodeling. Despite an increasing body of evidence on infectious and inflammatory mechanisms contributing to CF lung disease [20], host-derived factors that modulate CF lung disease severity and may serve as biomarkers are yet poorly defined. The CLP YKL-40 is induced at sites of inflammation, and previous studies suggested a role for this ancient glycoprotein as biomarker and potential therapeutic target in asthma [8], [18] and COPD [9]. It remained, however, elusive whether the accumulation of YKL-40 is specific for these disease conditions or whether YKL-40 increase reflects chronic pulmonary inflammation. In particular, the role of YKL-40 in CF lung disease has not been characterized so far. Here we demonstrate that YKL-40 protein levels are highly increased in the airways of patients and mice with CF lung disease and correlate with neutrophilic inflammation and airflow obstruction. Similar as in asthma, YKL-40 serum levels in CF patients were regulated genetically by promoter SNPs [11].

Previous studies found increased YKL-40protein levels and expression, respectively, in the circulation and lung from patients with severe asthma [8] and COPD [9]. Recently, YKL-40 has been described to play a role in hyperoxia-induced acute lung injury [10]. YKL-40 has also been implicated in a variety of non-pulmonary inflammatory diseases, such as rheumatoid arthritis [12], systemic sclerosis [13], myocardial infarction [14], meningitis[15], diabetes [16] and cancer[17]. We found that increased YKL-40 levels correlated with neutrophilic cellular infiltrate in CF patients. This finding may correspond to the results from Chupp et al. demonstrating that YKL-40 serum levels were particularly increased in severe asthma, a disease phenotype frequently featuring a more pronounced neutrophilic than eosinophilic airway infiltrate associated with corticosteroid resistance. Given this latter study and our present data, it remains to be investigated in future studies whether increased YKL-40 serum/airway levels are restricted to asthma subtypes with a pronounced neutrophilic inflammation.

The question arises which factors underlie the highly increased YKL-40 levels in CF patients. We speculate that YKL-40 levels in CF reflect the extent of neutrophilic airway inflammation rather than being modulated directly by the genetic CF defect (Cystic Fibrosis Transmembrane Conductance, CFTR). Neutrophils have been reported to store YKL-40 intracellularly in their secondary granules [21] and CF airway neutrophils were consistently found to express CD66b [23], indicating post-activation and release of secondary granules. YKL-40 protein levels in our study were highest in CF sputa, where numerous activated neutrophils accumulate, whereas in CF sera only moderately increased levels were found. At rising YKL-40 airway levels, the CLP may leak out into the circulation, explaining the increased levels found in serum of CF patients. Nevertheless, CFTR deficient neutrophils may intrinsically release more YKL-40 than control cells, a hypothesis that should be addressed in future studies.

The promoter SNP rs4950928 in the *YKL-40* gene was previously found to modulate YKL-40 serum levels and asthmatic lung disease severity [11]. Our studies demonstrated that two *YKL-40* SNPs (rs871799 and rs880633), located in the promoter region or exon 5, respectively, modulated age-adjusted lung function [19] in CF patients. Consistent with the previous study in asthmatics [11], we found that rs4950928 affected YKL-40 serum levels, but differently in our CF cohort, rs4950928 variants had no association with lung function in CF patients. Besides rs4950928, we identified a second promoter SNP (rs871799) that affected YKL-40 serum levels in CF patients. Based on previously published studies [11] and the herein described genetic studies, we suggest that at least two distinct promoter SNPs modulate YKL-40 serum levels in CF patients. We further speculate that rs871799 could be involved in both regulating YKL-40 serum levels and modulating FEV1, while rs880633 may affect YKL-40 functionality that, in turn, may modulate pulmonary CF disease (FEV1), without regulating YKL-40 expression and protein (serum) levels. Beyond that, the impact of *YKL-40* SNPs on lung function might depend on the type of pulmonary disease pathology (Th2-driven asthmatic versus CF lung disease). Within asthmatics, severe neutrophil-associated asthma may represent a distinct entity featuring increased YKL-40 levels [24], [25].

Despite significant processes in the field of chitinase-like proteins in the last years, including generation and characterization of transgenic mouse models, the biological and pathophysiological function of secreted YKL-40 in pulmonary diseases remains enigmatic [26]–[28]. Based on previous studies, using a genetic *brp-39* knock-out and a humanized YKL-40 transgenic overexpressing mouse, demonstrating that YKL-40/BRP-39 plays a role in tissue remodeling, cell death pathway regulation and airway obstruction [29],we speculate that released YKL-40 contributes to the cellular homeostasis and tissue remodeling, thereby modulating pulmonary function and inflammation in CF. However, additional studies focused on the functionality of YKL-40 in the CF microenvironment are required to understand the pathophysiological mechanisms linking YKL-40 and CF airway inflammation.

In summary, our studies demonstrated increased airway levels of the chitinase-like protein YKL-40 in CF patients compared to control individuals. This finding in CF patients could be corroborated by studies in a CF-like murine disease model. In both human and murine CF airway fluids, YKL-40/BRP-39 levels correlated with lung function, suggesting that increased YKL-40/BRP-39 levels may have an impact on airway obstruction in CF patients. These results suggest YKL-40 levels in sputum as potential biomarker of neutrophilic airway inflammation in CF.

## Supporting Information

Table S1 **CF genotyping patient cohort.** # no FEV1 data available for 20 patients; * predicted FEV1 values for CF patients at age of 20 years, as previously reported (19); § no microbiological data available for 18 patients.
(DOC)

Table S2 **Primers for genotyping** ***YKL-40*** **tagging SNPs.** dbSNP IDs (www.ncbi.nlm.nih.gov/SNP), SNP positions of genotyped SNPs (counted from the first nucleotide of the initiation codon as +1), and primers used for matrix-assisted laser desorption/ionization time-of-flight mass spectrometry (MALDI-TOF).
(DOC)

Methods S1 **Supplementary methods.**
(DOC)
