# Supplementary material for: The Chitinase-Like Protein YKL-40 Modulates Cystic Fibrosis Lung Disease
Source: PLoS One. 2011 Sep 20;6(9):e24399. doi: 10.1371/journal.pone.0024399 (PMC3176766; doi:10.1371/journal.pone.0024399)
Supplement: Table S1 — CF genotyping patient cohort. # no FEV1 data available for 20 patients; * predicted FEV1 values for CF patients at age of 20 years, as previously reported (19); § no microbiological data available for 18 patients. (DOC) [file pone.0024399.s001.doc]

**The chitinase-like protein YKL-40 modulates cystic fibrosis lung disease**

Andreas Hector^1,8^*, Michael S. D. Kormann^1,8^*, Ines Mack^8^*, Philipp Latzin^2^, Carmen Casaulta^2^, Elisabeth Kieninger^2^, Zhe Zhou^3^, Ali Ö. Yildirim^5^, Alexander Bohla^5^, Nikolaus Rieber^1,8^, Matthias Kappler^8^, Barbara Koller^6^, Ernst Eber^9^, Olaf Eickmeier^7^, Stefan Zielen^7^, Oliver Eickelberg^5^, Matthias Griese^8^, Marcus A. Mall^3,4^ and Dominik Hartl^1,8^**

^1^ Children’s Hospital and Interdisciplinary Center for Infectious Diseases, University of Tübingen, Tübingen, Germany

^2^Department of Paediatrics, University of Berne, Inselspital CH-3010 Berne, Switzerland

^3^Division of Pediatric Pulmonology & Allergy and Cystic Fibrosis Center, Department of Pediatrics III, University of Heidelberg, Heidelberg, Germany

^4^Translational Lung Reserach Center, University of Heidelberg, Heidelberg, Germany

^5^Comprehensive Pneumology Center, Institute of Lung Biology and Disease (iLBD) University Hospital, Ludwig Maximilians University and Helmholtz Zentrum München, Munich, Germany

^6^Department of Dermatology and Allergy, Ludwig-Maximilians-University, Munich, Germany

^7^Department of Pediatric Pulmonology, Allergy and Cystic Fibrosis, Children's Hospital, Frankfurt, Germany

^8^Research Center, Children’s Hospital, Ludwig-Maximilians-University, Munich, Germany

^9^Paediatric Department, Respiratory and Allergic Disease Division, Medical University of Graz, Austria

* equal contribution

** Correspondence:

Dominik Hartl

Children’ s Hospital and Interdisciplinary Center for Infectious Diseases

University of Tübingen, Germany

Hoppe-Seyler-Str. 1

72076 Tübingen, Germany

Phone: +49 – 7071 – 29 – 87199

FAX: +49 – 7071 – 29 – 5482

E-mail: dominik.hartl@med.uni-tuebingen.de

**ONLINE SUPPLEMENT**

**Supplementary Table S1.** CF genotyping patient cohort

|  | CF cohort (n=338) |
| --- | --- |
| Gender (female/male) | 164 / 174 |
| Age (mean ± SD) | 20.2 ± 10.2 yrs |
| FEV1 % of predicted (recent value) (mean ± SD) | 82.5 ± 29.0 ^#^ |
| FEV1@20 years* (mean ± SD) | 79.7 ± 23.5 |
| CFTR ΔF508 (homozygous/heterozygous/others) | 151 / 100 / 87 |
| Positive *Pseudomonas aeruginosa* microbiology | 196 ^§^ |
