# Supplementary material for: The Chitinase-Like Protein YKL-40 Modulates Cystic Fibrosis Lung Disease
Source: PLoS One. 2011 Sep 20;6(9):e24399. doi: 10.1371/journal.pone.0024399 (PMC3176766; doi:10.1371/journal.pone.0024399)
Supplement: Table S2 — Primers for genotyping YKL-40 tagging SNPs. dbSNP IDs (www.ncbi.nlm.nih.gov/SNP), SNP positions of genotyped SNPs (counted from the first nucleotide of the initiation codon as +1), and primers used for matrix-assisted laser desorption/ionization time-of-flight mass spectrometry (MALDI-TOF). (DOC) [file pone.0024399.s002.doc]

**The chitinase-like protein YKL-40 modulates cystic fibrosis lung disease**

Andreas Hector^1,8^*, Michael S. D. Kormann^1,8^*, Ines Mack^8^*, Philipp Latzin^2^, Carmen Casaulta^2^, Elisabeth Kieninger^2^, Zhe Zhou^3^, Ali Ö. Yildirim^5^, Alexander Bohla^5^, Nikolaus Rieber^1,8^, Matthias Kappler^8^, Barbara Koller^6^, Ernst Eber^9^, Olaf Eickmeier^7^, Stefan Zielen^7^, Oliver Eickelberg^5^, Matthias Griese^8^, Marcus A. Mall^3,4^ and Dominik Hartl^1,8^**

^1^ Children’s Hospital and Interdisciplinary Center for Infectious Diseases, University of Tübingen, Tübingen, Germany

^2^Department of Paediatrics, University of Berne, Inselspital CH-3010 Berne, Switzerland

^3^Division of Pediatric Pulmonology & Allergy and Cystic Fibrosis Center, Department of Pediatrics III, University of Heidelberg, Heidelberg, Germany

^4^Translational Lung Reserach Center, University of Heidelberg, Heidelberg, Germany

^5^Comprehensive Pneumology Center, Institute of Lung Biology and Disease (iLBD) University Hospital, Ludwig Maximilians University and Helmholtz Zentrum München, Munich, Germany

^6^Department of Dermatology and Allergy, Ludwig-Maximilians-University, Munich, Germany

^7^Department of Pediatric Pulmonology, Allergy and Cystic Fibrosis, Children's Hospital, Frankfurt, Germany

^8^Research Center, Children’s Hospital, Ludwig-Maximilians-University, Munich, Germany

^9^Paediatric Department, Respiratory and Allergic Disease Division, Medical University of Graz, Austria

* equal contribution

** Correspondence:

Dominik Hartl

Children’ s Hospital and Interdisciplinary Center for Infectious Diseases

University of Tübingen, Germany

Hoppe-Seyler-Str. 1

72076 Tübingen, Germany

Phone: +49 – 7071 – 29 – 87199

FAX: +49 – 7071 – 29 – 5482

E-mail: dominik.hartl@med.uni-tuebingen.de

**ONLINE SUPPLEMENT**

**Supplementary Table S2.** Primers for genotyping *YKL-40* tagging SNPs

| **dbSNP ID** | **Position** | **1st primer** | **2nd primer** |
| --- | --- | --- | --- |
| rs871799 | -14,120 | ACGTTGGATGGAGAGTGAATCTTTGGTGGC | ACGTTGGATGAACACATGCTGCCCTTAGTC |
| rs2153101 | -12,723 | ACGTTGGATGGGTCTTCATCCAGTGCCAAC | ACGTTGGATGTTGAAAGAAAGTGCCAGCTC |
| rs946263 | -9,630 | ACGTTGGATGTGGCCTTCCTAAGAAAAACC | ACGTTGGATGCTTTCTCACATGGTCATCAG |
| rs4950929 | -4,374 | ACGTTGGATGGCTTTACAAAGCCTCCAGAC | ACGTTGGATGTGTATGGCTAGCGAAACCAG |
| rs6691378 | -1,371 | ACGTTGGATGAGAGGTGGACAAAAAGTGGC | ACGTTGGATGTTGGGTACAAAAGGAGGCAG |
| rs10399805 | -247 | ACGTTGGATGCTATGGATTACCAGAGGAGG | ACGTTGGATGCATGAAGACCTGACCCAAAG |
| rs4950928 | -131 | ACGTTGGATGCCTTTATATACCTGTCCCAC | ACGTTGGATGTGACACATAGCTCAGTTCCC |
| rs1538372 | +1,220 | ACGTTGGATGGCAGACCAGTTTGTATGCAG | ACGTTGGATGCTGAGGTCTCTTGCCGAATC |
| rs880633 | +2,951 | ACGTTGGATGCTTTGATGGGCTGGACCTTG | ACGTTGGATGCTGACCAGCACCTTGATTAG |
| rs2275352 | +5,573 | ACGTTGGATGTCAGTACAGTCCTGCAAGAG | ACGTTGGATGAGACTTTGGGCCTCAGTTTC |
