# Supplementary material for: The Chitinase-Like Protein YKL-40 Modulates Cystic Fibrosis Lung Disease
Source: PLoS One. 2011 Sep 20;6(9):e24399. doi: 10.1371/journal.pone.0024399 (PMC3176766; doi:10.1371/journal.pone.0024399)
Supplement: Methods S1 — Supplementary methods. (DOC) [file pone.0024399.s003.doc]

**The chitinase-like protein YKL-40 modulates cystic fibrosis lung disease**

Andreas Hector^1,8^*, Michael S. D. Kormann^1,8^*, Ines Mack^8^*, Philipp Latzin^2^, Carmen Casaulta^2^, Elisabeth Kieninger^2^, Zhe Zhou^3^, Ali Ö. Yildirim^5^, Alexander Bohla^5^, Nikolaus Rieber^1,8^, Matthias Kappler^8^, Barbara Koller^6^, Ernst Eber^9^, Olaf Eickmeier^7^, Stefan Zielen^7^, Oliver Eickelberg^5^, Matthias Griese^8^, Marcus A. Mall^3,4^ and Dominik Hartl^1,8^**

^1^ Children’s Hospital and Interdisciplinary Center for Infectious Diseases, University of Tübingen, Tübingen, Germany

^2^Department of Paediatrics, University of Berne, Inselspital CH-3010 Berne, Switzerland

^3^Division of Pediatric Pulmonology & Allergy and Cystic Fibrosis Center, Department of Pediatrics III, University of Heidelberg, Heidelberg, Germany

^4^Translational Lung Reserach Center, University of Heidelberg, Heidelberg, Germany

^5^Comprehensive Pneumology Center, Institute of Lung Biology and Disease (iLBD) University Hospital, Ludwig Maximilians University and Helmholtz Zentrum München, Munich, Germany

^6^Department of Dermatology and Allergy, Ludwig-Maximilians-University, Munich, Germany

^7^Department of Pediatric Pulmonology, Allergy and Cystic Fibrosis, Children's Hospital, Frankfurt, Germany

^8^Research Center, Children’s Hospital, Ludwig-Maximilians-University, Munich, Germany

^9^Paediatric Department, Respiratory and Allergic Disease Division, Medical University of Graz, Austria

Running head: YKL-40 in CF

* equal contribution

** Correspondence:

Dominik Hartl

Children’ s Hospital and Interdisciplinary Center for Infectious Diseases

University of Tübingen, Germany

Hoppe-Seyler-Str. 1

72076 Tübingen, Germany

Phone: +49 – 7071 – 29 – 87199

FAX: +49 – 7071 – 29 – 5482

E-mail: dominik.hartl@med.uni-tuebingen.de

**ONLINE SUPPLEMENT**

**Supplementary Methods**

In total, 338 CF patients were included in this study. Only subjects, who regularly visited our CF care unit since at least 4 years were included in our studies. For 20 patients, no FEV1 data was available and therefore those patients were not included in the FEV1 association analyses. For the remaining 318 patients, estimated FEV1 values at age-adjusted 20 years were calculated from a minimum of five consecutive years of patient data. In total, we have included 13,256 FEV1 values into our longitudinal analyses for the 318 patients. One hundred fifty-one CF patients were ∆F508 homozygous, 100 were ∆F508 heterozygous carriers of the CFTR gene, and 87 had CFTR mutations other than ∆F508. One hundred ninety-six patients were positive for *P. aeruginosa* microbiology (bacteria isolated in at least 2 consecutive sputum samples with a minimum of a six-month interval). Genomic DNA was extracted from whole blood by a standard salting out method and DNA samples were genotyped using matrix-assisted laser desorption/ionization time-of-flight (MALDI-TOF) mass spectrometry (Sequenom, San Diego, CA, USA) as described in detail elsewhere (34). PCR assays and associated extension reactions were designed using the SpectroDESIGNER software (Sequenom). Specific primer sequences are given in **Supplementary table S2.** Derived genotype frequencies were compared with the expected allelic population equilibrium based on the Hardy-Weinberg equilibrium test to control for technical genotyping errors. All SNP data were based on the results of the National Institute of Health mutation screening program of innate immunity genes performed in a standard set of immortalized human samples from the Coriell Institute for Medical Research, Camden, New Jersey (ccr.coriell.org). Statistical p values for the SNPs were adjusted for multiple comparisons.
